# Assessment of the Miniature Kramer Shear Cell to Measure Both Solid Food and Bolus Mechanical Properties and Their Interplay with Oral Processing Behavior

**DOI:** 10.3390/foods9050613

**Published:** 2020-05-11

**Authors:** María Dolores Álvarez, Jaime Paniagua, Beatriz Herranz

**Affiliations:** 1 Institute of Food Science, Technology and Nutrition (ICTAN-CSIC), 28040 Madrid, Spain; 2 Pita López Foundation, 28400 Madrid, Spain; logocerebral@gmail.com; 3 Department of Food Technology, Veterinary Faculty, Complutense University, 28040 Madrid, Spain; herranzh@vet.ucm.es

**Keywords:** miniature Kramer shear cell, solid food, bolus, mechanical properties, texture, oral processing

## Abstract

This study assessed the usefulness of the miniature Kramer shear cell to determine relevant instrumental parameters of solid foods and bolus counterparts, examining their relationships with oral processing behaviors to obtain greater knowledge about the texture perception process. Six solid foods with different textural properties were tested. Bolus mechanical properties were also determined by means of cone penetration tests and rheological measurements, and their particle size distributions by sieving. Oral processing behavior (chewing time, number of chews, chewing rate, eating rate) and food saliva uptake (SU) of a young volunteer and a panel of 39 untrained participants were analyzed. The Kramer mechanical properties were very suitable for detecting different levels of food and bolus textural hardness and fracturability and the associated degrees of fragmentation achieved during mastication. Chewing time and number of chews were highly correlated with Kramer food and bolus mechanical properties for the single subject and for the panel’s oral processing behaviors. For the single subject, SU and eating rate also showed strong correlations with food and bolus mechanical properties, unlike chewing rate and food moisture content (FMC). In contrast, eating rate, FMC, and SU did not vary with the oral activities of the panel.

## 1. Introduction

Oral food processing is an essential, complex, dynamic process closely related to sensory perception, especially perception of food texture and flavor [1]. In turn, texture perception is a dynamic mechanism that depends on food properties and their changes during oral processing [2]. However, there is still a need for new strategies to quantify and better interpret textural results from instrumental measurements, and to achieve a better understanding of the relation between texture perception and fundamental mechanical parameters [3]. According to [3], for any given food it is essential, firstly, to ascertain its dominant textural attributes and the fundamental mechanical parameters to which they are primarily linked in order, secondly, to determine the parameters that are most important. Crispy/crunchy character is also an important sensory characteristic of some solid foods on which consumers base their appreciation, and in addition, crispness and crunchiness are acoustic attributes, and therefore the pitch of the sound emitted when the food is bitten or chewed is another characteristic aspect of both attributes. Crispy behavior likely implies that the energy dissipated due to viscoelasticity, as well as that due to friction processes are small, allowing high crack speeds and therefore the formation of shock waves with the speed of sound during fracture propagation [3].

Bourne [4] stated that objective tests measure real textural properties of materials, and are divided into fundamental, empirical, and imitative tests. Fundamental tests measure well-defined rheological properties, but they may not be very useful for measuring what is sensed in the mouth when food is masticated. Additionally, they are easier to apply with liquid or liquid-like products and in modeling of soft solid foods. For example, fracture stress and strain of cylindrical specimens of agar gels were determined by uniaxial compression, whereas *κ*-carrageenan/locust bean gum gels were tested by means of torsional fracture [5]. Furthermore, dynamic texture perception of emulsion-filled gels was found to be linked to fracture properties obtained from uniaxial compression tests [6]. However, most real hard solid foods fail to meet the requirements for application of fundamental tests. Hard solid foods do not have rheological properties that are independent of stress and strain conditions; moreover, they are anisotropic and heterogeneous, and it is very difficult to obtain test pieces or specimens with a uniform and regular shape from them. Empirical tests (puncture, shear, extrusion, etc.) measure parameters that are more poorly defined, but practical experience shows that they are often successful in measuring textural properties of foods [4,7]. Imitative tests, such as texture profile analysis (TPA) or the Kramer test, imitate the conditions to which the food material is subjected in practice, and closely duplicate mastication or other eating processes.

However, some researchers have recently been questioning the validity of TPA data [8,9], and they suggest looking for more rigorous mechanical testing approaches. Despite these recent criticisms, all the relationships previously found between either TPA or other empirical properties and oral processing behavior cannot be dismissed. Instrumental texture of five commercial ham samples was measured by using both TPA and Warner–Bratzler shear tests on cylindrical specimens, and the duration of mastication and number of chewing cycles through to swallowing were mainly related to TPA hardness, TPA chewiness, and Warner–Bratzler shear force [10]. Recently, Wee et al. [11] also used TPA to obtain objective measurements of textural attributes of rectangular or cylindrical specimens of a wide range of solid foods (*n* = 59), exploring relationships between instrumental textural properties, macronutrient composition, and eating behaviors. Although the authors compressed the foods to a 30% or <30% strain to avoid fracture, foods that were springy, cohesive, chewy, and resilient were associated with slower eating rates and more chews per bite. In addition, the authors indicated that variations in sample geometry and dimensions were an important limitation in their work as a consequence of the high degree of heterogeneity between foods. In another study, fracture mechanics of fifteen commonly consumed fruit, vegetable, and dairy food products were analyzed using the imitative Volodkevich bite jaws test [12], and the number of chew cycles and maximum oral residence time were significantly correlated with the maximum peak force, number of peaks, and gradient obtained by the Volodkevich test. Engelen et al. [13] also reported that harder and drier products require a higher number of chews before swallowing, although in that study they did not indicate how force and deformation were obtained. More recently, Krop et al. [14] also used Volodkevich and uniaxial puncture tests to determine hydrogel mechanical properties, finding that the fracture properties of the gels correlated well with number of chews and chewing duration, although they did not correlate with chewing rate. Although foods are not materials, and although properties such as force and energy cannot be considered intensive material properties [9], they have been shown to provide measures of food texture analogous to those experienced during oral processing.

Therefore, the future direction of research should be to try to apply empirical and imitative tests as rigorously as possible, but our knowledge of food texture perception and oral processing will not advance much if we keep trying to apply only fundamental tests to hard solid foods. As Szczesniak [15] said, fundamental tests are most useful to the food technologist as a way of providing a basis for the development of more meaningful empirical tests. In addition, efforts should also be directed to finding instrumental texture methods that offer universal coverage of foods under identical testing conditions.

Accordingly, a few years ago Stable Micro Systems (SMS, Ltd., Godalming, UK) developed the imitative miniature Kramer shear cell (HDP/MK05). To date, no studies have examined the food mechanical properties provided by this instrument, and their potential relationships with oral processing behavior. However, the miniature Kramer cell closely mimics the early stages of mastication, and it is possible to use larger or smaller sizes of very different samples, such as individual solid foods or composite, multi-particle, non-uniform, ready-to-eat meals, many thickened semisolid foods, and even food boluses. Nevertheless, there is little research that relates bolus mechanical parameters to dynamic texture perception.

The first aim of this work was to use a miniature Kramer shear cell to carry out an objective instrumental characterization of the mechanical properties of six solid foods (banana, apple, carrot, dices of cured ham, peanut, and potato chips) with very different dominant textural attributes, and of their bolus counterparts, and also to compare Kramer bolus mechanical properties with those obtained from either a cone penetration (CP) test or rheological measurements. A second aim was to investigate the potential relationships of both food and bolus Kramer mechanical parameters with food oral processing behaviors of a young healthy volunteer, who formed boluses for instrumental measurement, and of a panel of 39 untrained healthy participants.

## 2. Materials and Methods

### 2.1. Solid Food Samples

Canarian banana, Golden Delicious apple, packaged Nantesa carrot (Horticola ES-MA, S.L., Valladolid, Spain), dices of Serrano cured ham (Incarlopsa, Cuenca, Spain), fried peanut (Importaco Casa Pons, S.A., Valencia, Spain), and potato chips (Cyl Ibersnacks, S.L., Valladolid, Spain) were selected to be used in the present study, and they were acquired from a local supermarket (Mercadona, Madrid, Spain). Bananas and apples were purchased “just ripe.” Apples, carrots, and cured ham were stored in a domestic refrigerator at 4 °C, whereas bananas, peanuts, and potato chips were kept at room temperature. All the foods were continually replaced in order to carry out all the different analyses.

### 2.2. Methods

#### 2.2.1. Physical Measurements of Solid Foods

##### Moisture Determination

The moisture content of the foods studied (FMC), expressed in relation to fresh weight, was determined as follows:

FMC (%) = 100 ((FW–DW)/(DW))

where FW = fresh weight of the food and DW = dry weight of the food. DW was obtained after 48 h in an oven at 80 °C. At least five replicates were performed for each selected food.

##### Kramer Mechanical Properties

Mechanical properties of the foods were measured with a TA.HDPlus texture analyzer (Stable Micro Systems Ltd., Godalming, UK) provided with Texture Exponent software (version 6.1.13.0), and equipped with a 250 kg load cell. A Kramer shear test was conducted on each food sample using a miniature Kramer shear (HDP/MK05) cell with a 5-bladed head at a deformation rate of 2 mm s^−1^. The test was performed with a constant food sample volume fixed at ≈5.20 cm^3^, which corresponds to about half the volume that the bottom compartment of the cell can contain, and which is quite similar to the amount of food sample commonly introduced into the mouth for oral processing. From the force–distance curves, values of maximum force (FKMF), average force (FKAF), and work (FKW) required to shear food sample were obtained. At least five replications were performed for each food.

##### Acoustic Properties

Crispiness/crunchiness is a dominant mechanical textural attribute of raw carrot, peanut, and potato chips [16]. For this reason, the sounds emitted during Kramer tests of carrot, peanut, and potato chips were simultaneously recorded by an acoustic envelope detector (AED), as described elsewhere [17]. The AED tests were performed in a laboratory with no special soundproofing facilities at an ambient temperature of 22 ± 2 °C. In addition to the mechanical properties just mentioned, in crispy/crunchy solid foods the number of force peaks was also calculated for a drop in force greater than 1 N. The AED parameters calculated from the sound–distance curves were maximum sound pressure level (SPL_max_), number of sound peaks (drop in sound pressure level greater than 2 dB), and average drop-off.

#### 2.2.2. Physical Measurements of Bolus Counterparts

##### Kramer Mechanical Properties

Kramer mechanical properties of bolus counterparts were measured using the same texture analyzer and miniature HDP/MK05 cell mentioned above under identical conditions. The Kramer test was always performed on a constant fixed bolus volume (≈5.20 cm^3^). From the force–distance curves, maximum force (BKMF), average force (BKAF), and work (BKW) required to shear the boluses were obtained. At least five boluses of each food sample were obtained in vivo for Kramer bolus measurements.

##### Cone Penetration (CP) Mechanical Properties

CP mechanical properties of food bolus counterparts were also measured using the above-mentioned texture analyzer equipped with a TTC (Texture Technologies Corporation) spreadability rig (HDP/SR) consisting of a male perspex 90° cone probe and a matched female perspex cone-shaped product holder with a volume of 7.51 cm^3^ and height of 18 mm. The expectorated bolus was placed in the lower holder and the surface was levelled with a spatula. The male probe was displaced at a 2 mm s^−1^ deformation rate, forcing the bolus to flow outward at 45° between the male and female cone surfaces during the test. From CP tests, bolus consistency (BCPC), bolus consistency per gram (BCPC/g), bolus average force (BCPAF), and bolus spreadability or work (BCPW), defined as the area generated under the maximum force, were obtained from the force–distance curves. At least five boluses of each food were obtained in vivo for the CP measurements.

##### Small Amplitude Oscillatory Strain (SAOS) Rheological Properties

Small amplitude oscillatory strain (SAOS) tests were carried out using a rotational Kinexus pro rheometer (Malvern Instruments Ltd., Worcestershire, UK) equipped with a 40 mm parallel-plate geometry and a 1 mm gap (bolus volume of 1.26 cm^3^) to measure the rheological properties of banana, apple, and potato chip boluses, and with a 20 mm parallel-plate geometry and 1.5 mm gap (bolus volume of 0.942 cm^3^) to measure the rheological properties of carrot, cured ham, and peanut boluses. A solvent trap was used to minimize moisture loss during tests. After loading the sample, there was a 5 min waiting period to allow the bolus to recover and reach 37 °C. The temperature was controlled to within 0.1 °C by a Peltier element in the lower plate, which was kept at 37 °C. To determine the linear viscoelastic (LVE) range, strain amplitude sweeps were run at 1 Hz by varying the shear strain (*γ*) of the input signal from 0.01% up to 10%. Changes in storage modulus (*G*′, Pa), loss modulus (*G*″, Pa), complex modulus (G*, Pa), and loss factor (tan *δ* = *G*″/*G*′, dimensionless) were recorded. Critical (maximum) values of shear strain (*γ*_max_), shear stress (*σ*_max_), G*_max_, and tan *δ* were used to limit the LVE range according to Campo-Deaño and Tovar [18]. At least five boluses of each food sample were obtained in vivo for the measurement of rheological properties.

##### Particle Characterization by Granulometric Analysis

The granulometric analysis was performed by manual dry sieving as described previously [19], with slight modifications. The bolus was first poured onto a 0.4 mm aperture sieve, washed carefully under running water to eliminate saliva and allow the particles to spread, and dried with hot air. Then the dried bolus was poured onto a stack of 9 sieves with apertures of 10, 7.1, 6.3, 4, 2.5, 1.6, 1, 0.8, and 0.4 mm (Mecánica Científica, S.A., Madrid, Spain), and manually sieved with the help of a paintbrush, and the particles retained on each sieve were weighed. The results were expressed as cumulative curves, using the weight of the particles that dropped through each sieve. From each curve, the median particle size (d50), defined as the aperture of a theoretical sieve through which 50% of the weight of the fragmented food could pass, was determined for carrot, cured ham, peanut, and potato chips. At least three boluses per food were obtained in vivo for bolus particle size granulometric analysis.

#### 2.2.3. Bolus Formation and Oral Processing Characteristics

In this study, a healthy young male volunteer (26 years old) formed all the boluses for all the different instrumental analyses mentioned above. He signed a consent form and did not receive any compensation for participation. In order to carry out the physical measurements, the subject was asked to place a normal, comfortably sized portion of each food in his mouth and chew it as usual but without swallowing. Then, at the end of complete mastication, when the subject felt the need to swallow, the food bolus was expectorated into a container and transferred to the various cells used to measure it. At least five boluses were collected for Kramer and CP mechanical property measurements. In six separate sessions, three boluses per food sample were collected for particle size granulometric analysis. The boluses were always collected immediately before carrying out each instrumental replicate.

In separate sessions, additional boluses were collected from the trained volunteer to analyze his oral processing behavior for these foods. For this purpose, the chewing time at the end of mastication and the number of chews to reach complete mastication were recorded by the volunteer himself. Then, from the volunteer’s performances, the complete chew rate (masticatory efficiency) was obtained by dividing the total number of chews by the total oral exposure time of each food in the mouth (chews per s). The average eating rate was calculated by dividing the grams chewed by the total oral exposure time recorded after full mastication for each individual food (g min^−1^) in accordance with Wee et al. [11]. The amount of saliva uptake at the end of mastication was also estimated by subtracting the weight of each food sample provided to the subject from that of the expectorated bolus counterpart.

In order to examine the oral processing response of a group of healthy consumers with a wide age range to the same foods, the oral processing behavior for the selected foods (chewing time and number of chews) and the amount of saliva uptake were also evaluated by a panel of 39 untrained participants recruited among students and employees of the Institute of Food Science, Technology and Nutrition (ICTAN-CSIC). The group comprised 31 females and 8 males, with ages ranging between 21 and 65 years, and with good dental health and no chewing difficulties. They signed a consent form and did not receive any compensation for their participation. Testing was carried out in a sensory laboratory equipped with eight individual booths [20]. To analyze oral processing characteristics, weighed portions of each food were provided separately to the participants in random order, together with a chronometer. The participants were asked to chew each food sample normally, to expectorate the bolus after complete mastication just before swallowing, and to record the chewing time and count the number of chews required before spitting out each bolus into one of the containers provided. Each expectorated bolus was immediately weighed to determine saliva impregnation by weight difference as indicated above. The participants were provided with mineral water for rinsing between food samples, and they repeated the complete mastication process for each food as many times as they considered necessary. From the oral activities (chewing time and number of chews), the chewing and eating rates were also calculated.

As the healthy young male volunteer had a previous very different experience in forming boluses from those of the other 39 participants, in this study it was decided to analyze separately the results of his oral processing behavior instead of as part of a mean for comparison reasons.

### 2.3. Data Analysis

One-way analysis of variance (ANOVA) was performed to test for significant differences between mean instrumental mechanical properties of food samples and bolus counterparts, all the mean oral processing behaviors, and the mean saliva uptake values. The Tukey test was used for post hoc mean comparisons at a 95% significance level (*p* < 0.05). Pearson’s correlations were established between the instrumental analysis results and the two separate sets of parameters of oral processing behavior and saliva uptake values. Additionally, two categorical principal component analyses (CATPCA) were run to analyze the variability in instrumental mechanical properties of the food and bolus samples and the individual and mean oral activities and saliva uptake values from the masticatory performances of the individual volunteer and the group of 39 participants. Analyses were done using IBM SPSS Statistics for Windows, version 25.0 (IBM Corp., Armonk, NY, USA).

## 3. Results and Discussion

### 3.1. Instrumental Measurements

#### 3.1.1. Kramer Mechanical and Acoustic Properties of Foods

**Figure 1:**
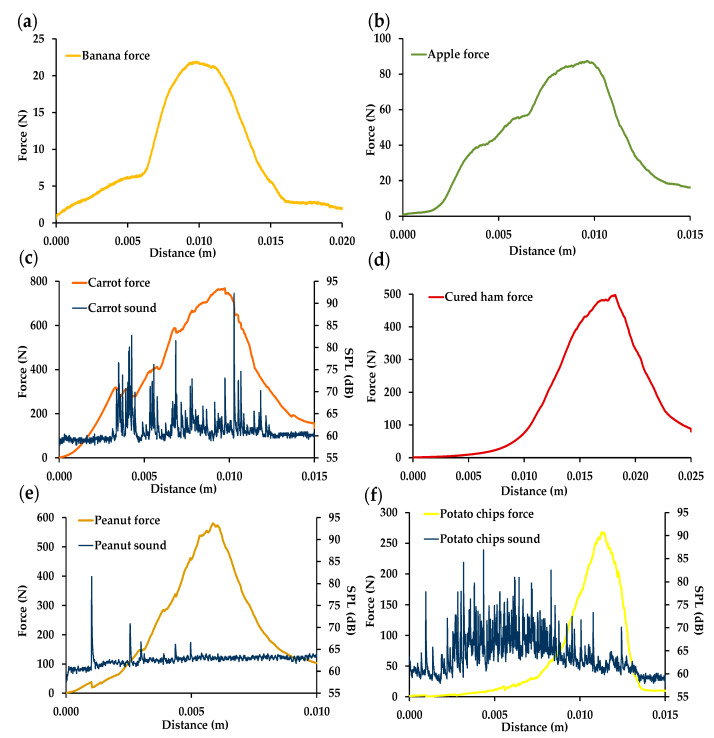
Typical miniature Kramer force–distance curves of selected foods: (**a**) banana; (**b**) apple; (**c**) carrot with sound pressure level (SPL)–distance curve; (**d**) cured ham; (**e**) peanut with SPL–distance curve; (**f**) potato chips with SPL–distance curve.

Figure 1 shows typical force–distance curves of the six foods upon combined compression, shearing, and extrusion triggered by the miniature Kramer shear attachment. Initially, pressing of the five blades on the food samples caused compression of microstructures as the load applied increased, and then the blades sheared through and extruded the samples before passing through a slotted plate. The test left all the food samples broken into small pieces, apparently in a similar degree of fragmentation to what occurs during oral processing. Each food sample had a force–distance curve with a characteristic shape that depended on its structural features. For example, carrot (Figure 1c), peanut (Figure 1e), and potato chip (Figure 1f) force–distance curves each had a jagged appearance due to fracture events associated with their different fracturability levels, and with the sound pressure level (SPL) curves recorded during their Kramer tests. Spikes of acoustic energy coinciding with the drops in the force curve could be seen in the case of carrot (Figure 1c), whereas in peanut (Figure 1e) the first major force peak was always accompanied by the greatest sound peak. In the case of potato chips (Figure 1f), numerous acoustic events were always recorded during the initial compression and packing prior to the shearing and extrusion of the sample. In ISO 5492 (2008) [16], raw carrot is mentioned as an example of a crunchy food with moderate fracturability, whereas peanut and potato chips are considered as brittle and crispy foods, respectively, both with high levels of fracturability.

The ability of solid foods to resist compression, shearing, and extrusion was expressed as the maximum and average forces (FKMF and FKAF) and absorbed energy or work (FKW) under the curve (Table 1). As a result of fixing the sample volume at 5.20 cm^3^, there were no significant differences between the weights tested for banana, carrot, and cured ham, whereas the weights of the apple and peanut samples were approximately 1 g lower, without significant differences between them. However, the weight of the potato chip samples was significantly lower than that of the other five foods tested, which was attributed to the high porosity of potato chips of around 70% [21].

Carrot required the highest FKMF and FKAF forces, followed by peanut and cured ham, potato chips, apple, and banana, in that order (Table 1). Carrot also needed the highest energy to be broken down, whereas the energy required for chewing peanut was half the energy needed for chewing cured ham. Therefore, in contrast with the force values, there were significant differences between the FKW values for cured ham and peanut, the former being almost double the value of the latter. Cured ham (Figure 1d) required a much greater distance than peanut (Figure 1e) to be fully extruded through the slotted plate, which was related to the structure of the meat tissue (connective tissue content, muscle fiber direction and diameters, etc.) [10]. This result was also associated with the low and high breakage functions of cured ham and peanut, respectively.

The AED parameters recorded during Kramer measurements of solid carrot, peanut, and potato chips are shown in Table 1. For these three foods, there was a good concordance between the number of force peaks and the number of sound peaks. In addition, there were no significant differences between the AED parameters obtained for carrot and potato chips, which reveals higher fracturability and loudness for both carrot and potato chips than for peanut. A higher number of force and sound peaks has been associated with greater sensory crispness [22]. However, as mentioned above, carrot is related to lower fracturability than both brittle peanut and crispy potato chips [16]. This discrepancy could be due to the fact that the mechanisms of sound production and the factors determining crispness are different in wet and dry foods. On the other hand, it is clear that food moisture content (FMC) does not influence the acoustic parameters (Table 1). In peanut and potato chips, with lower water contents, crispness has been reported to be primarily determined by the distribution of this low water content and the architecture of the product, whereas in carrot, with the highest moisture, the cell wall properties and turgor pressure play a more important role in determining crispness [23].

Otherwise, all the AED properties were higher for potato chips than for the peanut samples, confirming their higher reported fracturability (crispy vs. brittle) [16]. For potato chips, this mean SPL_max_ value was similar to those obtained for various commercial types of potato chips fractured by means of a penetration test with a spherical probe, with values ranging between 80.4 and 91.8 dB [22].

#### 3.1.2. Mechanical Properties of Boluses

The use of the Kramer cell to measure not only a solid food but also the corresponding bolus makes it possible to compare the mechanical properties required to fracture a particular food by imitating chewing without saliva with the properties that correspond to the production, by real oral processing, of a bolus that is cohesive and safe to swallow. Supplementary Materials Figure S1 shows the typical Kramer force–distance curves obtained with the miniature Kramer cell for each food bolus counterpart. In comparison with the force–distance curves shown in Figure 1, the shape of the food bolus force–distance curves varied to a greater or lesser extent, depending on the food type. Carrot and potato chip boluses (Supplementary Materials Figure S1c,f) had smooth curves without fracture events, indicating that in these foods fracturability was lost during chewing. However, peanut bolus samples (Supplementary Materials Figure S1e) had a first force peak (or bioyield point) before reaching maximum force, showing that peanut fragments retain a certain degree of fracturability or crispness until they are swallowed.

The CP test measures the ability of each bolus to resist compression and extrusion, and it was used to compare the effectiveness of the miniature Kramer and CP methods for measuring bolus mechanical properties. As an example, Figure S2 (Supplementary Materials) shows a typical spreadability or CP force–distance curve obtained for a peanut bolus sample. Similar but smoother curves were obtained for the other food bolus counterparts. In all the boluses, increasing the load applied increased the deformation, and the sample was forced to flow outward until the maximum force was reached. In the peanut bolus, even without applying shearing, it was also possible to observe small fracture events in the CP force–distance curve (Supplementary Materials Figure S2), again indicating the crispiness of the peanut fragments at the end of mastication.

Table 2 shows the food bolus mechanical properties obtained from the miniature Kramer and CP tests, which could be considered as instrumental swallowing thresholds for either food particle size or particle lubrication [13]. The authors in [13] observed that product characteristics, and to a lesser extent oral physiology, significantly affected the swallowing threshold, defined as the number of chewing strokes used before the food is swallowed.

The miniature Kramer measurements indicated that the cured ham bolus required the highest applied forces and absorbed the highest energy (significantly higher BKMF, BKAF, and BKW values), followed by carrot, peanut, potato chips, apple, and banana, in that order. In contrast, for the solid foods, the highest Kramer mechanical properties corresponded to carrot, followed by cured ham and peanut (Table 1). Interestingly, the CP applied forces seem to indicate that the cured ham bolus was structurally weaker and required lower BCPF and BCPAF forces than the carrot bolus (Table 2), whereas the BCPW values for the two boluses were not significantly different. The banana bolus had the highest spreadability (the lowest BCPW value), flowing more easily under cone probe penetration during testing. It has been reported that banana spreads through the mouth, being “mushed” between the tongue and the hard palate, and swallowed as a single bolus [24].

The degrees of structure and lubrication have been reported to be the main parameters influencing bolus mechanical properties [25]. From a comparison of the Kramer mechanical properties measured for each solid food with those determined for each lubricated bolus counterpart (Table 1 and Table 2, respectively) it is possible to estimate the degree of comminution or fragmentation that each food would still require after imitative mastication in the absence of saliva to form a swallowable bolus. Large differences were observed between all the food and bolus Kramer properties, and the degree of fragmentation depended on the product characteristics. For example, the BKMF values for the banana, apple, carrot, cured ham, peanut, and potato chip boluses (Table 2) were 20.7%, 9.63%, 27.2%, 45.3%, 6.64%, and 6.97%, respectively, of the FKMF values measured by testing the solid foods. Similarly, comparison of the food and bolus Kramer work values (FKW and BKW, respectively) showed that the initial degree of structuring of the banana, apple, carrot, cured ham, peanut, and potato chips was reduced by 81.5%, 93.6%, 72.0%, 63.0%, 92.2%, and 96.9%, respectively, to obtain safe-to-swallow bolus counterparts. Consequently, these results appear to indicate that oral processing of peanut and potato chips, with a lower FMC (Table 1) and higher fracturability [16], is associated with a much higher degree of fragmentation than that observed in cured ham or carrot. Therefore, both FMC and fracturability have an effect on the degree of fragmentation achieved during chewing. Similarly, the softer banana and apple samples seemed to exhibit intermediate destruction after oral processing, although it was clearly greater in the case of apple. Apple is reported to be moderately fracturable [16].

#### 3.1.3. Particle Size Characterization of Boluses

**Figure 2:**
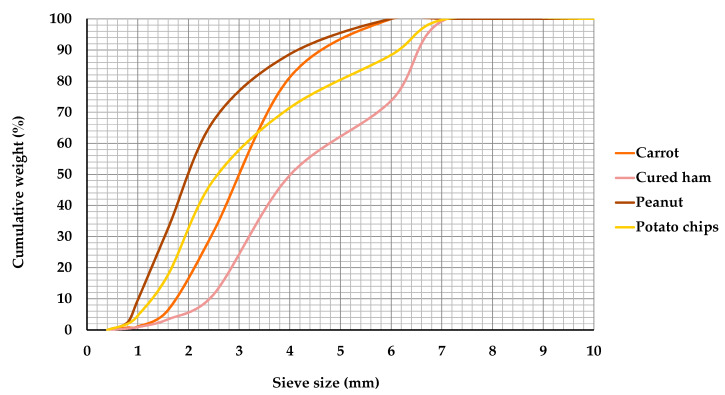
Particle size distribution curves of carrot, cured ham, peanut, and potato chip boluses collected at the end of in vivo mastication.

The decrease in degree of structure caused by fragmentation during mastication is usually quantified by determining the particle size distribution of the bolus fragments obtained by dry sieving, image analysis, or wet sieving, combined with laser diffraction for smaller particle sizes [10,25,26]. In this study, fragmentation was determined by dry sieving, although granulometric analysis was not appropriate for the banana and apple boluses because of their high stickiness, causing particle aggregation that impeded adequate spreading and sieving after washing and drying. This was probably due to the high fructose content of these two fruits. Figure 2 shows the mean particle size distribution curves of carrot, cured ham, peanut, and potato chip boluses expectorated by the volunteer at the point of swallowing.

There were larger particles in the cured ham bolus than in the carrot, potato chip, and peanut boluses, as illustrated by a shift of the curve towards higher particle size values. As an example, at the end of mastication, 38.2% of the particles in the peanut bolus were smaller than 1.6 mm, compared to less than 5% in the cured ham bolus. The degree of fragmentation, obtained as the median particle diameter (d50), was 4.04 ± 0.27, 3.02 ± 0.36, 2.62 ± 0.19, and 2.01 ± 0.13 mm for cured ham, carrot, potato chip, and peanut boluses, respectively. This result is in accordance with the degree of food destruction estimated above from comparison of the food and bolus Kramer properties, which was lower in cured ham and higher in peanut, respectively. Unfortunately, d50 values could not be determined for the apple and banana boluses. However, for carrots the d50 value was below 4.0 mm, which is considered as the upper limit of normal median particle size in a population of young persons with no masticatory disorders [27,28].

Previous measurements performed with the sieving method also indicated that the particles were much larger in vegetables than in nuts [29]. Moreover, the d50 values of peanut and carrot boluses expectorated after self-estimated complete mastication were 0.82 and 1.90 mm, respectively [30]. The authors in [30] observed that in 10 solid foods, the d50 value was a measure of the fracturability, with a small d50 corresponding to a marked fracture propagation in the food, resulting in numerous small particles. This is in agreement with the findings of the present work.

In addition, in carrot and peanut boluses, mean particle sizes of 0.780 ± 0.390 and 0.045 ± 0.039 mm^2^ were reported by Chen et al. [31], using image analysis. Rizo et al. [10] used image analysis to characterize the change of bolus particle size for five commercial cooked ham samples after 5, 10, and 15 chewing cycles (chewing test) and at the end of mastication, and also to calculate the median particle area (a50) at various mastication times. At the end of mastication, particle sizes between 10 and 50 mm^2^ constituted the most abundant fraction in the various cured ham boluses. For banana and apple boluses, d50 data have not been found in the literature. The distribution curve of the particle size obtained by means of laser diffraction showed two peaks for banana bolus [26], with particle sizes of 352.0 and 767.7 µm as the first and second most common, respectively. Recently, Bonnet et al. [32] reviewed the existing granulometry analyses of commonly consumed foods, including both peanut and carrot, in the literature, using the parameter d50, and provided useful tables to monitor mean d50 variations in future work. However, more research is needed to obtain the size and number of particles of banana and apple boluses by image analysis.

#### 3.1.4. Rheological Properties of Boluses

Amplitude sweeps were performed to investigate the LVE range of all the boluses collected at the end of mastication, where viscoelastic moduli are independent of stress. Table 3 shows the most representative magnitudes characterizing the extent of this LVE region for each bolus. There were significant differences (*p* < 0.05) between all the rheological properties of the food boluses. The critical *σ*_max_ and *γ*_max_ values provide information about structural stability and conformational flexibility, respectively [33].

Carrot bolus had the highest *σ*_max_ and the lowest *γ*_max_, which means that this physical network had the highest structural stability and the lowest flexibility, and it was also the most rigid (highest G*_max_ values) and the tightest. In contrast, cured ham bolus possessed the most flexible and least packed network, with the highest *γ*_max_ value, and this matrix also had intermediate rigidity and structural stability, as indicated by its medium G*_max_ and *σ*_max_ values. Moreover, the cured ham bolus also had the highest tan *δ* value (Table 3), indicating the lowest viscoelasticity, whereas apple and carrot boluses had the highest viscoelasticity (tan *δ* values closer to 0). However, tan *δ* values were lower than 0.5 in the six bolus samples, meaning that they behaved like a viscoelastic gel, as *G*′ was larger than *G*″, indicating the presence of a network structure [34].

Furthermore, although the apple and peanut boluses were quite flexible networks with similar *γ*_max_ values, peanut bolus was much more rigid than apple bolus. Finally, banana bolus was the least rigid network with the lowest stability. Unfortunately, there are no studies presenting comparable results of viscoelastic properties for boluses from solid foods, probably because the chewing process involves high deformations outside the LVE range. As indicated by Sukkar et al. [35], there is very little information about bolus viscoelastic rheological properties.

### 3.2. Oral Processing Behaviors

#### 3.2.1. Obtained from a Healthy Young Male Volunteer

Table 4 show the oral processing characteristics and the amount of saliva taken up by each food during the masticatory processes performed by the healthy young male volunteer, who was 26 years old. Cured ham required the significantly (*p* < 0.05) highest number of chews, whereas carrot bolus formation needed the highest oral exposure time. Because of its high number of strokes, cured ham also had the significantly highest chewing rate, whereas there were no significant differences between the chewing rates of the other foods. This result shows that, despite wide variation in chewing time and number of chews among foods, the chewing rate is very constant [36], regardless of the food mechanical properties. In fact, the chewing rates of hydrogels [14] were similar to those obtained for these hard solid foods, confirming previous findings regarding this oral activity parameter.

The lowest eating rate was associated with the complete mastication of potato chips, whereas banana and apple had significantly much more rapid eating rates than the other foods. In a study involving 47 different foods, potato chips were also eaten at a slower eating rate (7.1 g per minute) [36], and the ten “slowest” foods had lower moisture content. In addition, with the exception of potato chips, there is also a trend for increasing eating rate with decreasing Kramer mechanical properties of both foods and boluses (Table 2 and Table 3). Other authors, who considered TPA hardness and eating rate, observed the same trend in 59 solid foods [11]. Furthermore, the weight of each food sample before eating was always lower than that of each bolus counterpart, indicating that saliva was incorporated in all six foods to form boluses that were safe to swallow (Table 4). Nevertheless, there were significant differences in the moistening of the boluses at the swallowing point, with cured ham and apple presenting the highest and lowest saliva uptakes, respectively.

From the relationship between the initial weight of each food placed in the mouth and the saliva incorporated into each bolus, a saliva:food ratio of 1:12, 1:52, 1:5, 1:2, 1:4, and 1:1 was estimated to be required for banana, apple, carrot, cured ham, peanut, and potato chips, respectively, to form a cohesive bolus. Therefore, cured ham incorporated 26 times more saliva than apple, in agreement with their lower and higher saliva uptake values (Table 4), but required only half the saliva needed for potato chips to form a swallowable bolus. In foods, saliva incorporation has been stated to be a function either of moisture content or of structure [37], with dry foods generally requiring a large quantity of saliva. In this study, also, potato chips, which had the lowest water content and slowest eating rate, required the highest addition of saliva to produce a ready-to-swallow bolus, whereas the opposite was true for apple, which had the highest moisture content (Table 1). Therefore, for each food, insalivation would make up for the initial moisture content differences, as was also found for various cooked ham samples [10]. Forde et al. [36] suggested that the solid:water ratio probably drives either chewing time or eating rate. Indeed, according to other authors [38], the saliva:food ratios obtained reflect that the amount of saliva secreted per gram of food depends greatly on product characteristics such as water content. Moreover, the results also highlight the importance of making a careful and judicious choice of the saliva:food ratio to be used for each specific food in studies of simulated oral processing.

Correlations were also established between all the oral activity parameters of the healthy young volunteer (data not shown). Both oro-sensory exposure time and saliva uptake were significantly correlated positively with the number of chews, whereas eating rate was significantly correlated negatively with saliva impregnation. However, chewing rate did not correlate with the other mastication parameters, as observed previously [14].

#### 3.2.2. Obtained from 39 Healthy Untrained Volunteers

Table 5 shows the mean oral activity parameters obtained from the eating behaviors of 39 healthy participants with an average age of 41.5 ± 12.7 years. As an example, Table S1 in Supplementary Materials shows the individual oral activity parameters for potato chips together with the corresponding ages of the 39 participants. Curiously, there was less variation in the chewing time and number of chews for the six foods than when the young volunteer consumed the same food items. The average chewing time ranged between 10 s (banana, apple, and peanut) and 19 s (carrot and cured ham), with the average number of chews ranging from 11 (banana) to 25 (carrot and cured ham). Despite the wide variation in the ages of the participants, there was no significant difference between the average chewing rates of foods, which were all lower and in a narrower range than those obtained from the oral behavior of the young volunteer (Table 4). It has been stated that most people chew at a rate of 1.0–1.2 chews s^−1^, regardless of differences in food texture [36]. Cured ham, with a higher degree of structure and d50 value, and a moisture content of 58.1% (Table 1), had the slowest average eating rate, and, again, banana and apple the highest ones.

Mean saliva uptake values were negative for banana and apple, which indicated that there was no saliva incorporation into the bolus after full mastication of both banana and apple fruits (Table 5). This result could be due, at least partially, to the probability that some spontaneous swallowing occurred at the end of the mastication of these softer and wetter foods, especially as it was the first time that the participants had performed this task. In contrast, the amount of saliva incorporated into the bolus was higher in the case of potato chips, followed by cured ham. The relationships between the average oral activity parameters were also examined (data not shown). As observed with the oral activities of the young volunteer, chewing time was significantly positively correlated with the number of chews, whereas saliva uptake showed a very significant negative correlation with eating rate. Therefore, the participants chewed the foods that needed the incorporation of more saliva more slowly, confirming results from previous studies [11,36].

Consequently, despite the huge differences in the ages of the 39 participants, their eating behaviors were quite homogeneous, and the average oral processing behaviors were reasonably in line with the individual chewing behaviors of the young volunteer (Table 4). In addition, correlations were established between the individual and mean masticatory parameters in order to examine relationships between the two sets of eating behaviors (Supplementary Materials Table S2). Number of chews and oral residence time of the young volunteer showed a significant correlation with the same average oral parameters obtained from the 39 untrained participants. Additionally, eating rates and saliva incorporations were both significantly correlated between them, indicating that for these foods the chewing performances of a group of healthy dentate subjects with a wide age range was similar to that of a healthy young subject 26 years old. In contrast, high variability in masticatory parameters was observed in young adults (aged 18–25) with complete dentition and “normal” occlusion when chewing an artificial test food [39]. In the elderly, gradual and significant deterioration of the food bolus was observed and was associated with alterations in muscular force, production of saliva, and jaw or tongue motility [19].

### 3.3. Relation between Measured Physical Properties and Oral Processing Behaviors

Two CATPCA analyses were carried out to summarize the variations between foods regarding instrumental measurements and oral processing behaviors of the young volunteer and of the 39 participants. CATPCA presents several advantages over standard principal components analysis (PCA) in that it can handle nominal, ordinal, and numerical variables simultaneously and can deal with nonlinearities in the relationships between them [40].

**Figure 3:**
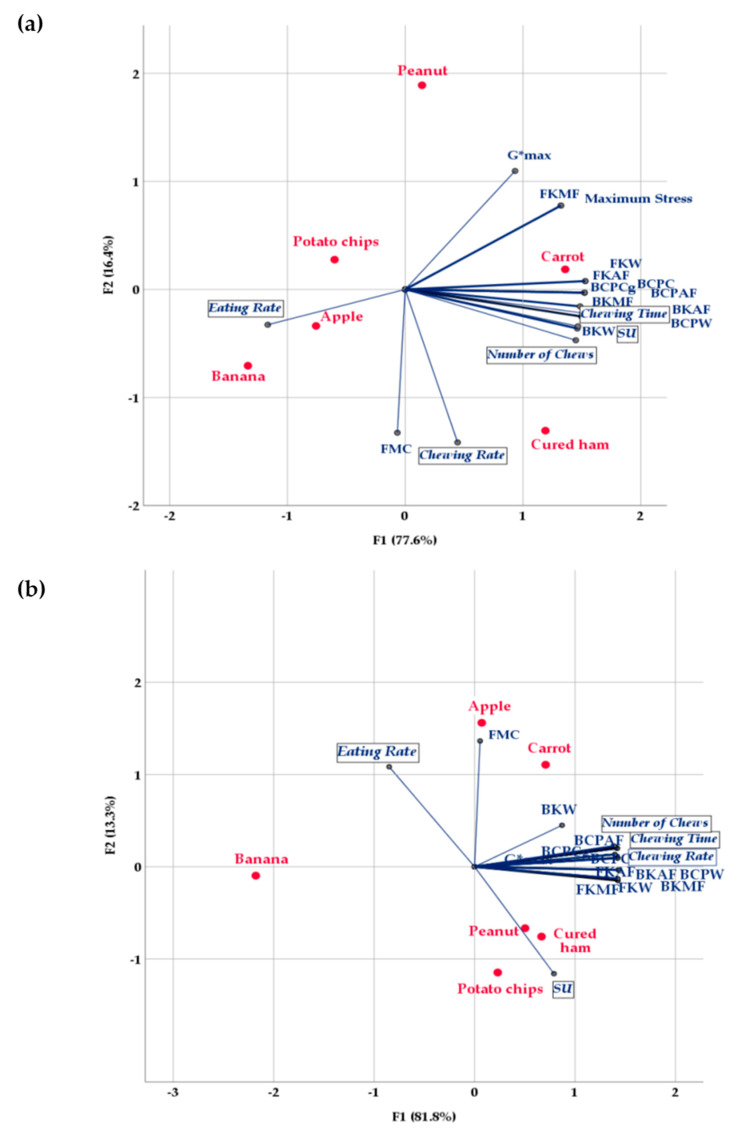
CATPCA plot showing variation in food products regarding instrumental measurements and oral processing behaviors: (**a**) of the young volunteer; (**b**) of the 39 untrained participants. Abbreviations are spelled out in Table 1, Table 2, Table 4, Table 5.

The first two dimensions explained 94.0% of the variability in the oral processing characteristics of the young volunteer (Figure 3a), and up to 95% in the mean oral activity parameters of the participating panel (Figure 3b). Figure 3 shows the relative positioning of the food samples and variables in the spaces generated by the two CATPCA analyses, revealing a different discrimination of the food set in the two groups in the analyses. As all the average values of the physical measurements were the same in the two CATPCAs; the results indicate a different contribution of food mechanical properties to the oral processing behavior of a single young subject or a group of subjects with various ages.

With regard to the individual eating behaviors of the single subject (Figure 3a), the first dimension explained 77.6% of the total variability and was mainly related to food Kramer mechanical properties, Kramer and CP bolus properties, oral exposure time, total number of chews, and saliva incorporation, all positioned on the right side. Engelen et al. [13] observed a high correlation (*r* = 0.913) between number of chew cycles and time at swallow regardless of food type, as food that remains in the mouth longer needs more chewing to form a swallowable bolus [12]. Some mechanical properties and eating behaviors overlap owing to their high correlation (Figure 3a). This first dimension was also negatively associated with eating rate, which appeared on the left side. The second dimension (16.4%) was related to chewing rate and moisture content (FMC), which were positioned in the negative part, and also to the bolus rheological property G*_max_, which was placed in the positive part of this dimension. Therefore, from this analysis it is possible to confirm that the eating rate seems to be mainly influenced by food properties, whereas the chewing rate seems to relate more to individual differences and eating capability [14]. Nevertheless, the variables that were not well correlated with the first two dimensions were eating rate, followed by FMC, G*_max_, and chewing rate, in that decreasing order. FKMF, FKAF, FKW, and maximum stress were the mechanical properties best explained by the two dimensions.

The first dimension explained almost 82.0% of the total variability in the analysis of the mean eating behaviors of the panel (Figure 3b). In this case, the first dimension also presented positive correlations with instrumental food and bolus properties, and with chewing time, number of chews, and chewing rate. The second dimension placed moisture content and eating rate in the positive part and saliva uptake (SU) in the negative one, indicating that a higher degree of moisture in the initial food structure implies either a faster eating rate or a lower quantity of saliva incorporation. With the exception of BKW, in general the first two dimensions provided a good explanation of all the variables in this analysis.

With regard to how the food samples were positioned in the corresponding two-dimensional spaces, Figure 3a shows that the first dimension separated banana, apple, and potato chips on the left side from carrot and cured ham on the right side. Indeed, the first three foods had lower oral exposure time and number of chews (Table 4), lower food and bolus mechanical properties (Table 1, Table 2 and Table 3), and in the case of banana and apple faster eating rate and lower SU (Table 4). On the other hand, carrot and cured ham had significantly higher mechanical properties and longer chewing time, and required a greater number of chews to achieve an adequate particle size for swallowing. Peanut, at the top of the second dimension, was separated from the other foods by its lower moisture content (Table 1) and chewing rate (Table 4). Cured ham, in the bottom right part of the plot, was differentiated from the other foods by having the highest chewing rate. Therefore, the first dimension provided a good explanation of the positions of the food samples in the space, and the food and bolus mechanical properties had a more significant effect in the food discrimination observed than the individual oral activity characteristics.

However, in the CATPCA analysis of the average oral processing behaviors of the 39 participants, the position of the samples in the space was explained better by the second dimension (Figure 3b). In this analysis, only banana was placed on the left side of the first dimension, owing to its low mechanical and rheological properties and its low chewing time, number of chews, and chewing rate (Table 5). However, the second dimension clustered apple and carrot together in the positive part because of their higher moisture contents, whereas cured ham, peanut, and potato chips were placed in the negative part of the second dimension, mainly because they required more saliva incorporation and had slower eating rates (Table 5). In this second analysis, oral processing behaviors influenced the food positioning in the two-dimensional space more than physical properties, and the food mechanical properties affected the oral processing behaviors more, probably because of the high variation between the individual eating behaviors of the 39 subjects.

These results also seem to corroborate other previous findings. Interestingly, the CATPCA of the eating behaviors of the young volunteer (Figure 3a) also shows that FMC did not correlate either with food mechanical properties or with SU. In this case, FMC was only correlated with chewing rate, and therefore moisture content hardly contributed to discrimination of the foods in that two-dimensional space. This result seems to indicate that the degree of moisture of the initial food structure did not contribute significantly to the food oral processing of the healthy young volunteer, who probably had a high bite force. The relation between food structure, food oral processing, human strength, and perception of difficulty has been studied [12]. In accordance with the authors just cited, food structure does significantly affect food oral processing (number of chew cycles and time in mouth), and it is correlated with the difficulty perceived. However, in a young population they did not find any relationship between the perception of difficulty and their individual oral force or chewing behavior. In the present study, lubrication by food moisture content correlated with neither the average eating rate nor the SU of the 39 participants (Figure 3b), which was also probably related to individual variations in eating capabilities and physiology. Xu et al. [41] recently reviewed the present knowledge about the effect of age-dependent changes on the quantity and quality of saliva in healthy elderly individuals, which in turn affect the individual saliva incorporation requirements for each food. In addition, for dry, highly fracturable biscuits, mechanical strength had a limited influence on the number of chewing cycles, whereas the amount of saliva secretion was a more decisive factor [12], as found in this study for both peanut and potato chips (Figure 3b).

## 4. Conclusions

Six solid foods with different dominant textural attributes and their bolus counterparts were characterized by using a miniature Kramer shear cell, and each food showed a different ability to resist compression, shearing, and extrusion because of its specific structural features. The degree of comminution or fragmentation of each food after imitative mastication without saliva was estimated by comparison of all the food and bolus Kramer properties and it was in agreement with the results obtained by sieving. In both solid foods and boluses, the degree of fragmentation depended on food moisture content and fracturability. Moreover, high correlations were found between these Kramer food and bolus mechanical properties and some oral processing parameters (chewing time and number of chews) of both a single subject and a group of 39 subjects’ oral processing behaviors, their chewing performances being all reasonably similar. Therefore, use of the miniature Kramer shear cell is recommended in order to acquire greater knowledge about dynamic perception of the texture of solid or soft foods, as with this cell it is possible to obtain objective mechanical properties of solid foods and their boluses and determine potential correlations with oral processing parameters. This could be very useful in the management of dysphagia, making it easier to obtain objective classifications of modified textures suitable for patients with dysphagia in order to ensure safe swallowing.

## Figures and Tables

**Table 1:** Mechanical and acoustic properties of foods obtained by using the miniature Kramer shear cell and food moisture contents.

Food	Food Weight (g)	FKMF (N)	FKAF (N)	FKW (J)	Force Peaks	SPL_max_ (dB)	Sound Peaks	Average Drop-off	FMC (%)
Banana	6.44a (0.134)	23.4d (1.53)	8.08c (0.591)	0.195e (0.017)	-	-	-	-	81.4b (2.91)
Apple	5.28 b (0.217)	83.8d (4.87)	31.1c (0.450)	0.779d (0.011)	-	-	-	-	89.1a (0.324)
Carrot	6.42a (0.390)	725a (42.3)	377 a (24.5)	5.65a (0.368)	25.0a (3.58)	90.0a (5.50)	24.0a (3.65)	7.94a (0.814)	92.2a (0.009)
Cured Ham	6.30a (0.141)	554b (71.4)	201b (21.0)	5.03b (0.525)	-	-	-	-	58.1c (1.95)
Peanut	5.52b (0.130)	574b (16.0)	200b (23.8)	2.48c (0.310)	13.0b (1.79)	80.6b (3.97)	12.0b (1.67)	6.11b (0.823)	4.65d (1.17)
Potato Chips	1.24c (0.182)	244c (0.091)	36.8c (3.63)	0.921d (0.091)	26.0a (3.00)	86.9a,b (1.75)	25.0a (3.36)	7.25a,b (0.450)	5.12d (0.352)

Mean value (standard deviation, SD). Values followed by the same letter within each instrumental parameter indicate no significant differences (*p* ≤ 0.05). FKMF: food Kramer maximum force; FKAF: food Kramer average force; FKW: food Kramer work; SPL_max_: maximum sound pressure level; FMC: food moisture content.

**Table 2:** Mechanical properties of boluses obtained by using the miniature Kramer shear cell and the TTC spreadability rig.

Food	BKMF (N)	BKAF (N)	BKW (J)	BCPC (N)	BCPC Per Gram (N g^−1^)	BCPAF (N)	BCPW (J)
Banana	4.84 d	1.79d	0.036 c	5.56 e	0.823 e	0.937e	0.006 c
	(0.528)	(0.092)	(0.002)	(0.534)	(0.118)	(0.080)	(0.000)
Apple	8.07 d	3.35 c,d	0.050 c	68.2 d	11.4 d	5.33 d	0.045 c
	(0.972)	(0.489)	(0.007)	(3.88)	(0.646)	(0.582)	(0.003)
Carrot	197 b	63.3 b	1.58 b	478 a	82.9 a	33.2 a	0.341 a
	(16.8)	(3.42)	(0.086)	(29.3)	(8.57)	(3.14)	(0.042)
Cured Ham	251 a	84.6 a	1.86 a	265 b	38.9 b	27.0 b	0.360 a
	(31.1)	(13.1)	(0.288)	(30.1)	(3.22)	(1.97)	(0.039)
Peanut	38.1 c	12.8 c	0.194 c	135 c	21.3 c	10.8c	0.133 b
	(4.79)	(0.821)	(0.015)	(17.0)	(3.83)	(0.706)	(0.006)
Potato Chips	17.0 c,d	2.85 c,d	0.029 c	24.1 e	11.6 d	3.41 d,e	0.042 c
	(0.972)	(0.311)	(0.003)	(2.26)	(1.42)	(0.213)	(0.003)

Mean value (standard deviation, SD). Values followed by the same letter within each instrumental parameter indicate no significant differences (*p* ≤ 0.05). BKMF: bolus Kramer maximum force; BKAF: bolus Kramer average force; BKW: bolus Kramer work; BCPC: bolus consistency; BCPC per gram: bolus consistency per gram; BCPAF: bolus average force; BCPW: bolus spreadability.

**Table 3:** Rheological properties of boluses (limit values of linear viscoelastic (LVE) range) obtained from amplitude sweeps.

Food	*σ*_max_ (kPa)	*γ*_max_ (%)	G*_max_ (kPa)	tan *δ*
Banana	0.006 e	0.251 c	2.44 c	0.285 a,b
	(0.001)	(0.001)	(0.548)	(0.016)
Apple	0.029 e	0.159 d	18.2 c	0.177 d
	(0.006)	(0.001)	(3.58)	(0.010)
Carrot	0.222 a	0.100 e	222 a	0.197 c,d
	(0.008)	(0.001)	(9.45)	(0.005)
Cured Ham	0.135 c	1.00 a	13.4 c	0.292 a
	(0.005)	(0.001)	(0.550)	(0.023)
Peanut	0.175 b	0.158 d	111 b	0.216 c
	(0.021)	(0.000)	(12.9)	(0.002)
Potato Chips	0.080 d	0.395 b	20.2 c	0.255 b
	(0.010)	(0.000)	(2.45)	(0.004)

Mean value (standard deviation, SD). Values followed by the same letter within each instrumental parameter indicate no significant differences (*p* ≤ 0.05). *σ*_max_: maximum shear stress amplitude; *γ*_max_: maximum shear strain amplitude; G*_max_: maximum complex modulus; tan *δ*: loss factor (= *G*″/*G*′).

**Table 4:** Oral processing behavior and bolus saliva uptake of a healthy young male volunteer for the selected foods.

Food	Chewing Time (s)	Number of Chews	Chewing Rate (chews s^−1^)	Eating Rate (g min^−1^)	Food Weight (g)	Bolus Weight (g)	Saliva Uptake (SU) (g)
Banana	9.20 d	13 d	1.42 b	47.8 a	7.29 a	7.88 b	0.588 c,d
	(0.837)	(1.00)	(0.136)	(4.18)	(0.133)	(0.456)	(0.493)
Apple	9.40 d	15 d	1.59 a,b	40.6 b	6.29 b	6.41 c	0.120 d
	(1.34)	(1.48)	(0.106)	(4.66)	(0.442)	(0.797)	(0.016)
Carrot	31.2 a	45 b	1.44 b	14.3 c	7.42 a	9.02 a	1.60 b
	(1.48)	(1.30)	(0.052)	(0.979)	(0.213)	(0.316)	(0.124)
Cured Ham	27.8 b	48 a	1.74 a	13.5 c	6.26 b	9.29 a	3.03 a
	(1.10)	(3.05)	(0.173)	(0.498)	(0.119)	(0.382)	(0.390)
Peanut	20.4 c	28 c	1.38 b	16.1 c	5.47 c	7.00 b,c	1.53 b
	(1.14)	(1.79)	(0.096)	(0.763)	(0.202)	(0.887)	(0.716)
Potato Chips	8.60 d	13 d	1.46 b	12.6 c	1.80 d	3.13 d	1.33 b,c
	(0.548)	(1.14)	(0.068)	(0.442)	(0.136)	(0.153)	(0.214)

Mean value (standard deviation, SD). Values followed by the same letter within each oral activity parameter indicate no significant differences (*p* ≤ 0.05).

**Table 5:** Mean oral processing behavior and bolus saliva uptake of 39 healthy participants for the selected foods.

Food	Chewing Time (s)	Number of Chews	Chewing Rate (chews s^−1^)	Eating Rate (g min^−1^)	Food Weight (g)	Bolus Weight (g)	Saliva Uptake (SU) (g)
Banana	10.0 b	11 b	1.16 a	43.8 a	6.90 b	6.78 a	−0.121 b,c
	(3.76)	(4.70)	(0.319)	(14.0)	(1.96)	(1.84)	(1.40)
Apple	10.6 b	14 b	1.34 a	49.9 a	8.19 a	7.74 a	−0.479 c
	(4.06)	(5.25)	(0.263)	(18.2)	(2.69)	(2.97)	(1.73)
Carrot	19.8 a	25 a	1.29 a	14.2 b	4.48 c	4.99 b	0.518 a,b
	(6.87)	(10.6)	(0.313)	(4.81)	(1.68)	(2.01)	(1.58)
Cured Ham	19.3 a	25 a	1.29 a	8.50 b	2.36 d	3.58 c	1.22 a
	(8.06)	(10.5)	(0.280)	(3.80)	(0.562)	(1.04)	(0.723)
Peanut	10.1 b	12 b	1.30 a	10.0 b	1.53 d	2.29 d	0.759 a
	(3.43)	(3.94)	(0.398)	(3.82)	(0.394)	(0.850)	(0.882)
Potato Chips	11.0 b	13 b	1.27 a	11.4 b	1.87 d	3.15 c,d	1.28 a
	(3.97)	(4.33)	(0.348)	(4.82)	(0.436)	(1.11)	(1.00)

Mean value (standard deviation, SD). Values followed by the same letter within each oral activity parameter indicate no significant differences (*p* ≤ 0.05).

## Supplementary Materials

The following are available online at https://www.mdpi.com/2304-8158/9/5/613/s1: Figure S1. Typical Kramer force–distance curves of food bolus counterparts collected at the end of mastication before swallowing; Figure S2. Typical cone penetration (CP) force–distance curve of peanut bolus counterpart collected at the end of mastication before swallowing; Table S1. Oral processing behavior and bolus saliva uptake of the 39 participants for potato chips; Table S2. Correlation matrix between individual (of the healthy young male volunteer, identified with (1)) and mean (of the 39 healthy participants with ages ranging between 21 and 65 years, identified with (2)) oral processing behaviors.
