# Supplementary material for: Assessment of the Miniature Kramer Shear Cell to Measure Both Solid Food and Bolus Mechanical Properties and Their Interplay with Oral Processing Behavior
Source: Foods. 2020 May 11;9(5):613. doi: 10.3390/foods9050613 (PMC7278702; doi:10.3390/foods9050613)
Supplement: Supplementary file 1 [file foods-09-00613-s001.pdf]

Table S1. Oral processing behavior and bolus saliva uptake of the 39 participants for potato chips.

Gender	Age	Chewing Time (s)	Number of Chews	Chewing Rate (chews s^{-1})	Eating Rate (g min^{-1})	Saliva Uptake (g)
Male	65	7.00	7	1.00	18.0	-0.500
Female	23	9.16	12	1.31	15.7	-0.100
Female	36	10.6	16	1.51	9.60	1.20
Female	39	8.00	9	1.13	17.3	1.80
Female	52	9.00	9	1.00	8.00	1.90
Male	60	12.0	12	1.00	12.0	0.600
Female	34	15.4	14	0.910	9.36	2.00
Male	22	15.0	19	1.27	8.02	3.80
Female	46	7.90	17	2.15	14.4	0.200
Male	39	8.20	11	1.34	13.9	0.200
Male	50	8.00	12	1.50	15.0	2.20
Female	30	16.9	20	1.19	7.12	1.50
Female	47	17.5	18	1.03	7.89	1.00
Female	44	14.0	20	1.43	9.00	2.30
Female	41	4.85	7	1.44	30.9	1.10
Female	60	15.0	14	0.933	9.60	1.10
Female	25	10.0	15	1.50	11.4	0.100
Female	63	6.41	9	1.40	15.9	0.600
Male	23	9.00	14	1.56	11.3	1.10
Female	38	6.00	9	1.50	11.0	1.70
Male	23	9.45	9	0.952	14.0	3.80
Female	29	7.88	12	1.52	18.3	1.50
Female	59	10.3	21	2.04	11.1	2.60
Female	57	11.8	11	0.932	11.7	2.00
Female	22	17.0	20	1.18	6.72	1.00
Female	37	14.0	16	1.14	8.14	0.800
Female	59	16.6	15	0.903	7.22	1.30
Female	49	14.0	12	0.857	4.71	1.50
Female	36	21.1	20	0.950	7.41	1.50
Male	44	6.80	9	1.32	12.4	1.00
Female	44	11.1	10	0.901	8.65	1.40
Female	39	7.50	10	1.33	12.0	1.00
Female	53	8.00	16	2.00	8.25	-0.600
Female	35	8.15	13	1.60	11.8	-0.200
Female	49	6.70	9	1.34	14.3	1.80
Female	33	16.0	22	1.38	4.50	1.00
Female	41	10.0	7	0.700	12.6	0.200
Female	50	8.00	13	1.63	10.5	1.70
Female	21	14.8	10	0.675	4.86	2.70
**Panel Mean**	41.5	11.0	13	1.27	11.4	1.28
**(SD)**	(12.7)	(3.97)	(4.33)	(0.348)	(4.82)	(1.00)

Mean value (standard deviation, SD). Values followed by the same letter within each oral activity parameter indicate no significant differences ($p \leq 0.05$).

Table S2. Correlation matrix between individual (of the healthy young male volunteer, identified with -(1)) and mean (of the 39 healthy participants with ages ranging between 21 and 65 years, identified with -(2)) oral processing behaviors. Significant values are in bold.

	**Chewing Time-(2)**	**Number of Chews-(2)**	**Chewing Rate-(2)**	**Eating Rate-(2)**	**Saliva Uptake-(2)**
**Chewing Time-(1)**	**0.879***	**0.868***	0.293	-0.627	0.402
**Number of Chews-(1)**	**0.919****	**0.916****	0.307	-0.615	0.439
**Chewing Rate-(1)**	0.482	0.548	0.363	-0.013	0.146
**Eating Rate-(1)**	-0.532	-0.523	-0.438	**0.961****	**-0.888***
**Saliva Uptake-(1)**	0.718	0.701	0.135	**-0.829***	**0.820***

Levels of significance are indicated with one asterisk (*) for $p \leq 0.05$ and two asterisks (**) for $p \leq 0.01$.

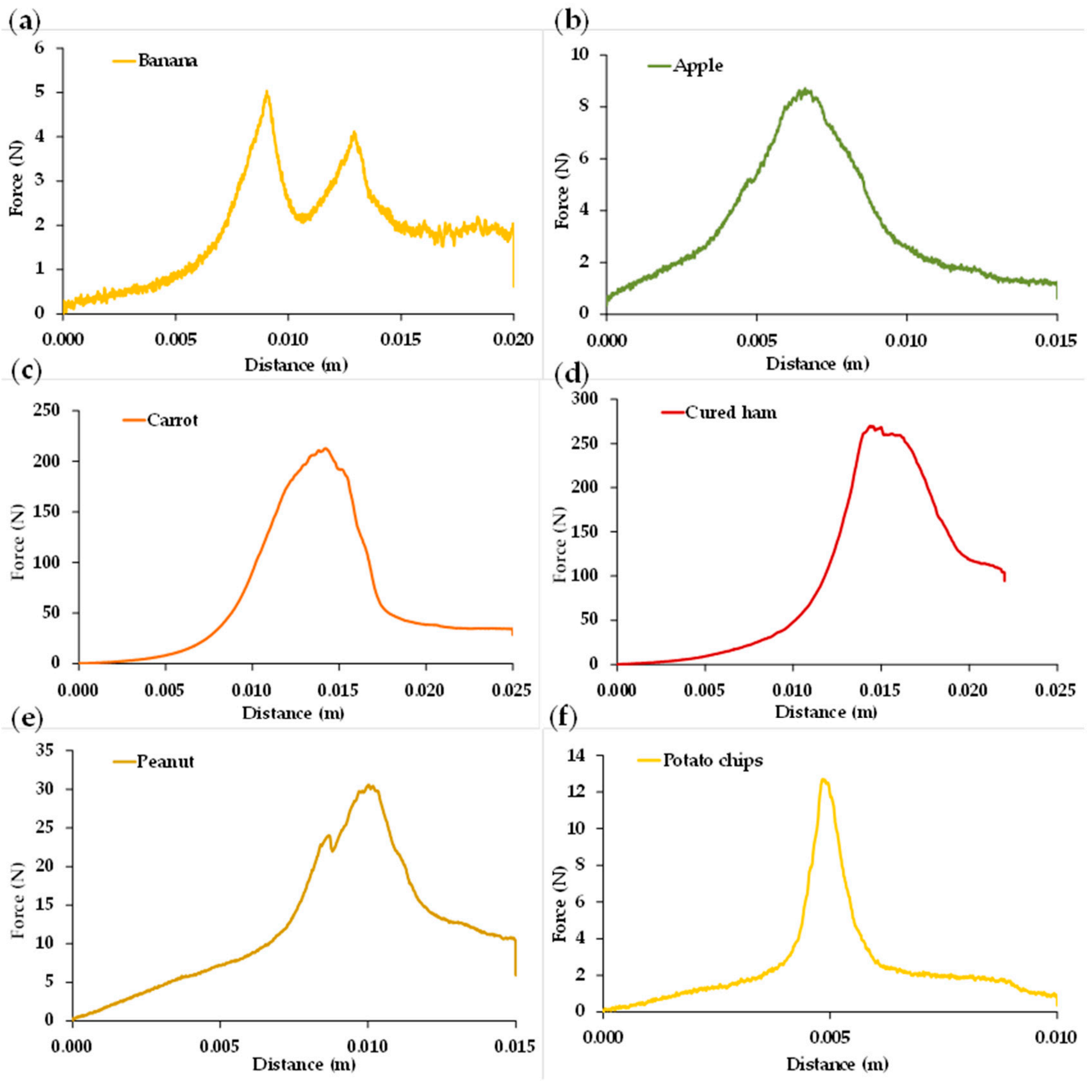

Figure S1. Typical Kramer force–distance curves of food bolus counterparts collected at the end of mastication before swallowing: (**a**) Banana; (**b**) Apple; (**c**) Carrot; (**d**) Cured ham; (**e**) Peanut; (**f**) Potato chips.

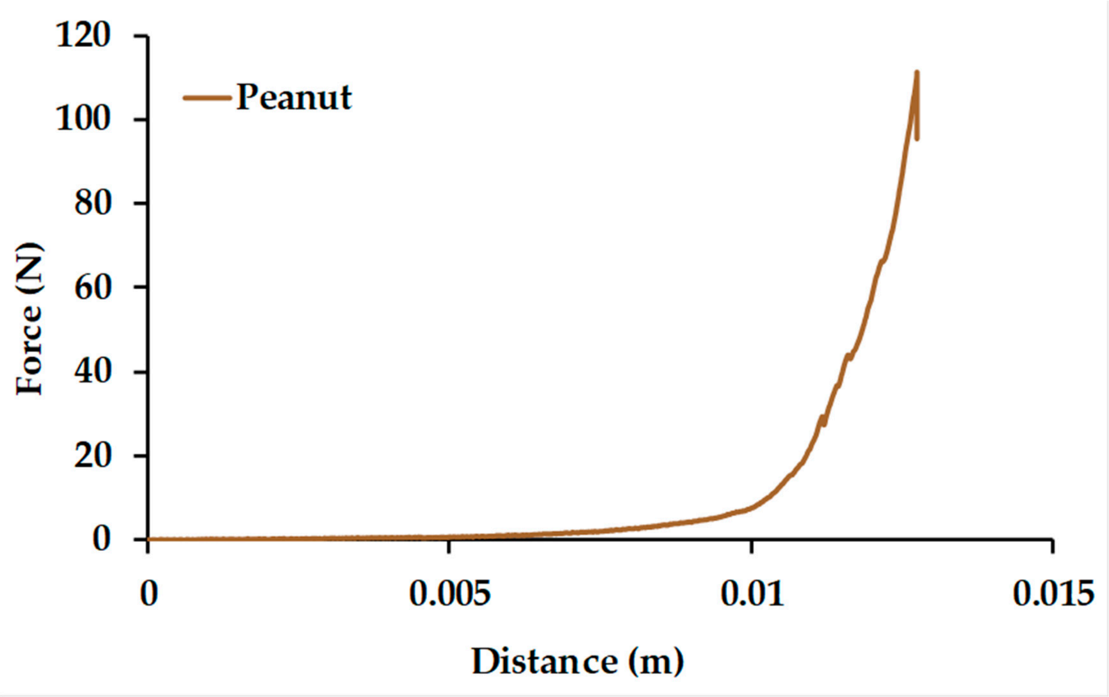

Figure S2. Typical cone penetration (CP) force–distance curve of peanut bolus counterpart collected at the end of mastication before swallowing.
